# The Synergistic Effects of Pyrotinib Combined With Adriamycin on HER2-Positive Breast Cancer

**DOI:** 10.3389/fonc.2021.616443

**Published:** 2021-05-21

**Authors:** Chaokun Wang, Shuzhen Deng, Jing Chen, Xiangyun Xu, Xiaochen Hu, Dejiu Kong, Gaofeng Liang, Xiang Yuan, Yuanpei Li, Xinshuai Wang

**Affiliations:** ^1^ Henan Key Laboratory of Cancer Epigenetics, Cancer Hospital, The First Affiliated Hospital, College of Clinical Medicine, Medical College of Henan University of Science and Technology, Luoyang, China; ^2^ Medical College, Henan University of Science and Technology, Luoyang, China; ^3^ Department of Internal Medicine, UC Davis Comprehensive Cancer Center, University of California Davis, Sacramento, CA, United States

**Keywords:** HER2 positive breast neoplasm, pyrotinib, adriamycin, synergistic, Akt

## Abstract

Pyrotinib (PYR) is a pan-HER kinase inhibitor that inhibits signaling *via* the RAS/RAF/MEK/MAPK and PI3K/AKT pathways. In this study, we aimed to investigate the antitumor efficacy of pyrotinib combined with adriamycin (ADM) and explore its mechanisms on HER2^+^ breast cancer. We investigated the effects of PYR and ADM on breast cancer *in vitro* and *in vivo*. MTT assay, Wound-healing, and transwell invasion assays were used to determine the effects of PYR, ADM or PYR combined with ADM on cell proliferation, migration, and invasion of SK-BR-3 and AU565 cells *in vitro*. Cell apoptosis and cycle were detected through flow cytometry. *In vivo*, xenograft models were established to test the effect of PYR, ADM, or the combined therapy on the nude mice. Western blotting was performed to assess the expression of Akt, p-Akt, p-65, p-p65, and FOXC1. The results indicated that PYR and ADM significantly inhibited the proliferation, migration, and invasion of SK-BR-3 and AU565 cells, and the inhibitory rate of the combination group was higher than each monotherapy group. PYR induced G1 phase cell-cycle arrest, while ADM induced G2 phase arrest, while the combination group induced G2 phase arrest. The combined treatment showed synergistic anticancer activities. Moreover, PYR significantly downregulated the expression of p-Akt, p-p65, and FOXC1. In clinical settings, PYR also exerts satisfactory efficacy against breast cancer. These findings suggest that the combination of PYR and ADM shows synergistic effects both *in vitro* and *in vivo*. PYR suppresses the proliferation, migration, and invasion of breast cancers through down-regulation of the Akt/p65/FOXC1 pathway.

## Introduction

Breast cancer (BC) is the most common malignant tumor among women in the world, with 2.1 million new patients and 626,679 deaths in 2018 (1). The overexpression of HER2 or gene amplification accounts for about 15-20% of all BC cases, which is related to the invasiveness of the tumor, and the prognosis is worse without appropriate therapy (2, 3). HER2, encoded by oncogene ErbB2, is a transmembrane protein in human cells (4), which is involved in regulating cell proliferation, differentiation, and apoptosis through the activation of signal transduction by homo- or hetero-dimerization (5). Therefore, blocking the HER2 pathway is considered a potential therapy for BC. At present, several HER2-targeted agents are available to treat HER2-overexpressing BC. Five drugs were approved by the U.S. Food and Drug Administration (FDA) for the treatment of HER2-positive BC, known as trastuzumab, pertuzumab, TDM-1, lapatinib, and neratinib (6–10). Furthermore, the Chinese State Drug Administration recently authorized a new tyrosine kinase inhibitor (TKIs), pyrotinib, for the treatment of patients with HER2-positive recurrence and metastasis breast cancer (11).

Pyrotinib is an oral, irreversible pan-ErbB receptor TKI with activity against HER1, HER2, and HER4 (12). Previous studies have suggested that pyrotinib can irreversibly inhibit multiple ErbB receptors and effectively inhibit the proliferation of HER2-overexpressing BC cells *in vivo* and *in vitro* (13, 14). By covalently binding with the ATP binding sites of intracellular kinase regions, pyrotinib inhibits the formation of homo- or hetero-dimerization and auto-phosphorylation of the HER family, thus blocking the activation of the RAS/RAF/MEK/MAPK and PI3K/AKT signaling pathways. AKT is activated *via* several mechanisms such as recruitment to the membrane by PIP3, PDK1, and mTORC2 (15–17). Activated phospho-AKT can phosphorylate a number of proteins including GSK-3b, 6-phosphofructo-2-kinase, and IκB (18, 19). The phosphorylation of IκB frees NF-κB and allows it to translocate to the nucleus to bind and subsequently activate target genes (20). The best characterized subunit of NF-κB is p65. This heterodimer is a potent activator of gene expression, where p65 is responsible for this activation (21–23). It was previously shown that activation of the NF-κB can upregulate FOXC1 expression. Overexpressed FOXC1 has been demonstrated in many different types of cancers (24–28). Recent studies have suggested that upregulation of FOXC1 may exacerbate cell invasion and indicate a poor prognosis due to EMT and drug resistance (24, 29, 30). It is unknown whether combination with adriamycin treatment inhibited BC cell proliferation, migration, and invasion through down-regulation of the Akt/p-65/FOXC1 signaling pathway.

In HER2^+^ cancers, the usefulness of pyrotinib has been shown in preclinical and clinical reports (31–35). However, the clinical efficacy of pyrotinib alone is limited, and it is anticipated that development trends will involve combining it with chemotherapy for anti-HER2 treatments in the future. Currently, several ongoing clinical trials are exploring the combination of anti-HER2 agents with chemotherapy in HER2-positive BC and the clinical benefit of combined therapy of pyrotinib and chemotherapy is expected (36).

Adriamycin, also known as doxorubicin, is an anthracycline antibiotic that functions by intercalating DNA and inhibiting topoisomerase II. It has been widely used in the combination therapy of first-line antitumor agents and other antitumor agents for breast cancer (37–39). Until now, there was no more powerful evidence for the combined effect of pyrotinib and chemotherapy. Therefore, we hypothesize that the combination of both drugs would show synergistic anticancer activities against HER2-positive BC. In the present study, we investigated the antitumor efficacy of pyrotinib in combination with adriamycin and explored its related mechanisms as new therapeutics for HER2-positive BC. These results suggest that the combination of pyrotinib and adriamycin show strong synergistic antitumor effects both *in vitro* and *in vivo*.

## Materials and Methods

### Cell Lines and Cell Cultures

HER2-positive breast cancer cell lines SK-BR-3 and AU565 cells were maintained in DMEM medium supplemented with 10% FBS. The cells were cultured at 37°C in a humidified atmosphere with 5% CO_2_.

### Chemicals and Antibodies

PYR was acquired from Hengrui Medicine Co. Ltd. ADM was acquired from Pfizer. Drugs were dissolved to a concentration of 200μg/ml in dimethyl sulfoxide (DMSO), diluted with PBS, and then stored at -80°C until use. The following antibodies: Akt, p-Akt, p65, p-p65, GAPDH, FOXC1 were obtained from Abcam (Abcam Trading Co., Ltd, Shanghai, China).

### Cell Viability Assay

Cell viability was assayed by the MTT assays. SK-BR-3 or AU565 cells were seeded in 96-well plates at a density of 3000–5000 cells/well and were treated for 12 h with PBS, PYR, ADM, or both drugs in combination. The treatments continued for 48 h. The MTT solution (0.1 mg/ml) was added and then cultured for another 4 h, and the medium was subsequently removed. Next, 150 μl of DMSO was added to dissolve the formed formazan crystals. The absorbance of each well was measured at 570 nm by a microplate reader (Bio-Tek, Norcross, GA, U.S.A.). The mean IC50 values were calculated by SPSS. CompuSyn (ComboSyn Inc.) was used to calculate the combination index (CI) values (40).

### Wound Healing Assay

Wound-healing assay was used to analyze the migration ability of SK-BR-3 and AU565 cells. We plated 1x10^6^ cells/well in 6-well plates and cultured them overnight until the cells reached 90% confluence. A straight scratch was created by a sterile pipette tip. The destroyed cells were rinsed off gently with PBS 3 times and followed by incubation with PBS, PYR (0.3, 3 μg/ml), ADM (0.1, 0.3μg/ml) or both drugs in combination (PYR 3 μg/ml + ADM 0.3μg/ml) for 24 h. Cell migration was observed and imaged at 0 h and 24 h with a digital camera. ImageJ software (NIH, Bethesda, MA, U.S.A.) was used to quantitatively analyze cells migrated to the denudated regions of each petri dish in the field of view. The experiments were independently performed three times.

### Transwell Invasion Assays

The aperture of the bottom membrane of the Transwell chambers or wells (Corning Inc., Corning, NY, USA) was 8 μm. The chambers were coated with Matrigel (Sigma-Aldrich, St. Louis, MO, USA), and were used for detecting the cell invasive ability. SK-BR-3 and AU565 cells were harvested and resuspended in serum-free DMEM, and 200 μl of cell suspension (5 × 10^5^ cells/ml) containing PBS, PYR (0.3, 3 μg/ml), ADM (0.1, 0.3μg/ml) or both drugs in combination(PYR 3 μg/ml + ADM 0.3μg/ml). The cells were cultured in an incubator at 37˚C with 5% CO_2_ for 24 h. The cells on the upper surface of the membrane were removed with cotton swabs. The migrated or invaded cells were fixed in 95% ethanol, stained with Hematoxylin. Cell numbers were counted in ten randomly selected fields under a light microscope at × 100 magnification. The cell numbers were counted by ImageJ software.

### Cell Cycle Analysis

For cell cycle arrest assay, SK-BR-3 and AU565 cells were starved for 24 h before treatments. Then cell were treated with PBS, PYR (0.3, 3 mg/ml), ADM (0.1, 0.3mg/ml) or both drugs in combination (PYR 3 mg/ml + ADM 0.3mg/ml). After treatment for 24 h, The treated cells were washed with PBS and fixed in darkness at -20°C with 70% pre-cooled ethanol for 1h. Then the fixed cells were washed with PBS and treated with RNase I at 37°C for 30 minutes. Finally, the cells were stained with PI at 4°C for an additional 30 minutes and measured by BD FACS caliber. Each experiment was independently repeated in triplicate.

### Apoptosis Analysis

SK-BR-3 and AU565 cells (2.5×10^5^ cells/well) were plated in 6-well plates for the apoptosis assay and treated with PBS, PYR (0.3, 3 μg/ml), ADM (0.1, 0.3μg/ml), or both drugs in combination (PYR 3 μg/ml + ADM 0.3μg/ml) for 48h. Then, cells were washed with PBS, adjusted to 1×10^6^ cells/ml, and treated with Annexin V-FITC/PI apoptosis detection kit based on the manufacturer’s protocol. Finally, the cells were detected by BD FACS caliber, using the BD CellQuest Pro software for analysis.

### Animals and Tumor Model

Female nude mice (4-5 weeks old) were raised in a specific pathogen-free animal facility (Temperature, 20-26°C; humidity, 40-60%; 12/12-h light/dark cycle; free access to food and water). The back of each mouse was subcutaneously inoculated with SK-BR-3 cells (1 × 10^7^) suspended in 0.2 mL PBS. When the tumor volume reached nearly 60mm^3^, mice were randomly assigned into 4 groups. Mice in each group (*n*=6) were treated *via* daily oral gavage with PYR (30 mg/kg/d), intravenous injection ADM (5 mg/kg/w) every week, or a combination of both drugs for 27 days. The weight and tumor size were measured twice a week. Tumor volume was calculated as *V*= (length × width^2^)/2. All of the animal experiments conformed to the requirements of the ethics committee.

### Western Blotting

The cells were treated with 0.3, 3, 10μg/ml of pyrotinib or 0.1, 0.3, 3μg/ml of adriamycin for 48h. Then, the cells were harvested and prepared for cytosolic and nuclear protein extraction using a cytoplasmic and nuclear protein extraction kit (Cowin Bio., Beijing, China) according to the manufacturer’s instructions. Equal amounts of protein were employed in 12% SDS-PAGE (Cowin Bio., Beijing, China) followed by transfer to PVDF membrane. After blocking with 5% skim milk powder in 0.1% Tween in phosphate buffered saline (PBST) at room temperature for 2 h, the membrane was incubated with primary antibody (Akt, p-Akt, p65, p-p65, FOXC1) at 4°C overnight. Subsequently, secondary antibodies were incubated at 37°C for 1 h. Finally, the membranes were washed with PBST and detected in Tanon 2500 chemiluminescence imaging system (Tanon, Shanghai, China). Image J software (NIH, Bethesda, MA, U.S.A.) was used for density analysis and quantitative analysis of protein level.

### Clinical Application of Pyrotinib

To evaluate the efficacy of PYR treatment, we retrospectively screened the breast cancer patient who was given PYR treatment, collected clinical data, chest computerized tomography (CT) scans, and paraffin-embedded pathological specimens. This study was approved by the Ethics Committee of the First Affiliated Hospital of Henan University of Science and Technology.

### Statistical Analysis

Statistical analysis was performed using IBM SPSS 23.0 software (SPSS, Chicago, IL, USA). All the data were presented as mean value ± SD, and statistically significant differences between different experimental groups and control groups were examined using one-way analysis of variance (ANOVA). *P*<0.05 was considered a statistically significant difference.

## Results

### Effects of Pyrotinib and Adriamycin on the Proliferation of Breast Cancer Cells

**Figure 1:**
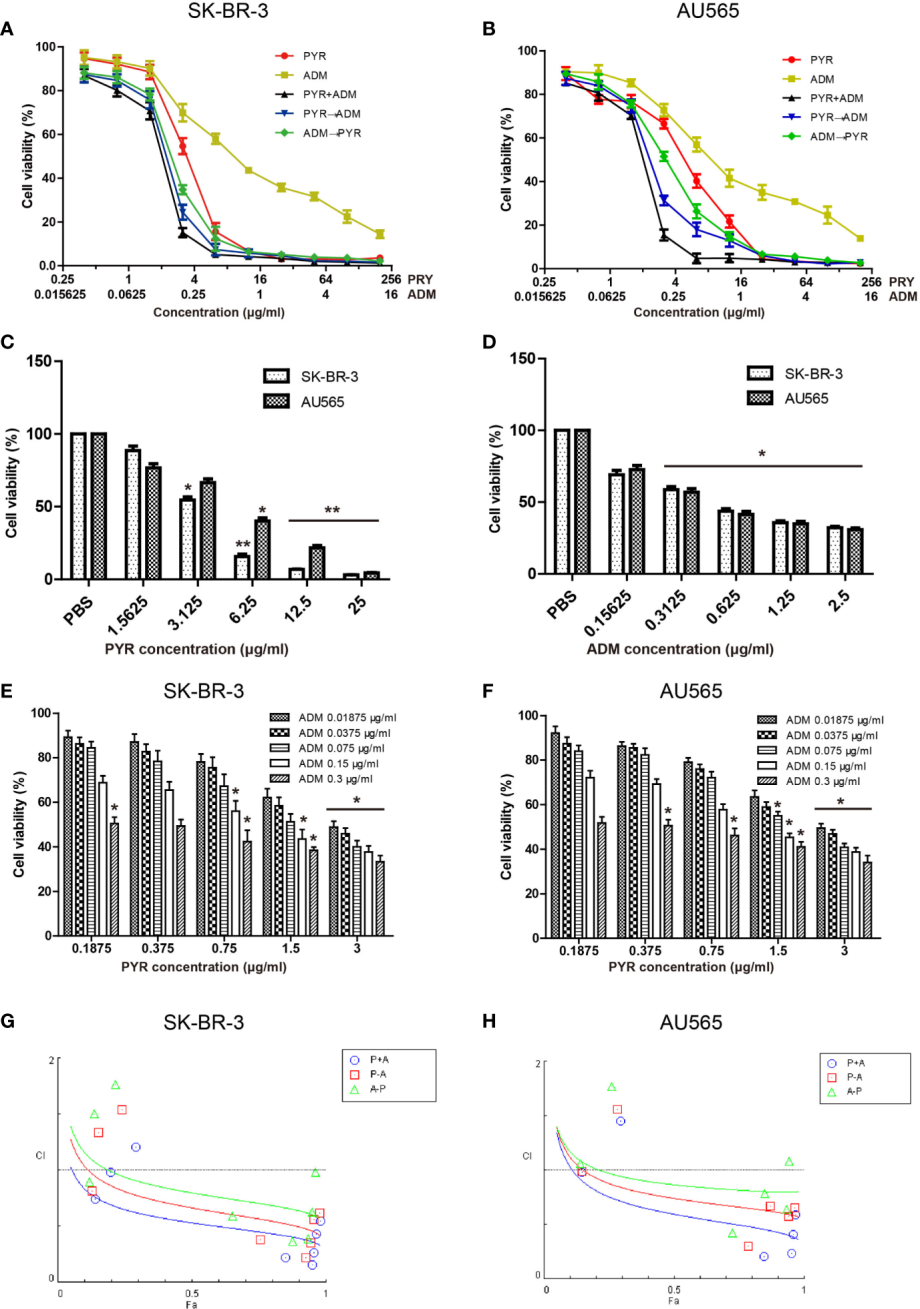
Effects of PYR and ADM on the viability of breast cancer cells. Proliferation activity of SK-BR-3 or AU565 cells was determined by the MTT assay after incubation for 48h with different concentrations of PYR or ADM. **(A, B)** SK-BR-3 or AU565 cells were treated with PYR or ADM alone or in combination or in sequences (PYR first for 6 h followed by ADM or PYR first for 6 h followed by ADM). **(C, D)** The histogram represents the statistical analysis for SK-BR-3 or AU565 cells were treated with PYR or ADM alone. **(E, F)** Proliferation activity of SK-BR-3 or AU565 cells was determined after incubation for 48h with different concentrations of PYR combination with 0.3, 0.15, 0.075, 0.0375, 0.01875μg/ml of ADM. **(G, H)** The combination index (CI) v.s. fraction affected (Fa) affected plot was calculated by Compusyn and depicted the combination effects. Synergistic growth inhibitory effects of PYR combined with ADM on SK-BR-3 or AU565 cells. Synergy is defined as CI values < 1.0, antagonism as CI values > 1.0, and additivity as CI values=1.0. *p<0.05 and **p<0.01 compared with the PBS group.

To assess the cytotoxicity of pyrotinib and adriamycin on SK-BR-3 and AU565 cells, cell viability was detected by MTT assays after treatment with various concentrations of pyrotinib or/and adriamycin for 48 h. As shown in **Figure 1**, we demonstrated that SK-BR-3 and AU565 cell growth was significantly inhibited at concentrations ranging from 3 to 200μg/ml of pyrotinib and 0.3 to 10μg/ml of adriamycin, and the inhibitory effect was positively correlated with the pyrotinib or adriamycin concentration. The mean IC_50_ values of pyrotinib for SK-BR-3 and AU565 cells were 3.03μg/ml, 3.82μg/ml. The mean IC_50_ values of adriamycin for SK-BR-3 and AU565 cells were 0.31μg/ml, 0.62μg/ml. Different concentrations of pyrotinib (0.3 and 3μg/ml) and adriamycin (0.1 and 0.3μg/ml) were used for the remaining experiments. To determine the synergistic antitumor effects, the combination index for SK-BR-3 and AU565 cells were calculated using the method of Chou and Talalay. As shown in **Figures 1G, H**, **Tables 1** and **2**. The results showed that co-treatment with pyrotinib and adriamycin was more effective in SK-BR-3 and AU565 cells.

**Table 1:** Combination Index Value and Fraction Affected of PYR and ADM on AU565 cells.

PYR c(μg/ml)	ADMc(μg/ml)	PYR+ADM		PYR-ADM		ADM-PYR	
		Fa	CI	Fa	CI	Fa	CI
0.39063	0.01953	0.12968	0.81322	0.12968	0.81322	0.11942	0.89319
0.78125	0.03906	0.15348	1.33928	0.15348	1.33928	0.13879	1.50464
1.5625	0.07813	0.24210	1.54313	0.24210	1.54313	0.21765	1.76330
3.125	0.15625	0.75557	0.37870	0.75557	0.37870	0.65233	0.59323
6.25	0.3125	0.92628	0.22036	0.92628	0.22036	0.87669	0.36236
12.5	0.625	0.94279	0.34830	0.94279	0.34830	0.93604	0.38605
25	1.25	0.95460	0.56432	0.95460	0.56432	0.94900	0.62706
50	2.5	0.97698	0.61692	0.97698	0.61692	0.96100	0.98412

Synergy is defined as CI values < 1.0, antagonism as CI values > 1.0, and additivity as CI values = 1.0.

**Table 2:** Combination Index Value and Fraction Affected of PYR and ADM on AU565 cells.

PYRc(μg/ml)	ADMc(μg/ml)	PYR+ADM		PYR-ADM		ADM-PYR	
		Fa	CI	Fa	CI	Fa	CI
0.39063	0.01953	0.14822	0.97918	0.13676	1.09062	0.12551	1.22266
0.78125	0.03906	0.20782	1.12981	0.17232	1.24982	0.16783	1.43563
1.5625	0.07813	0.29695	1.45174	0.27342	1.64753	0.26393	1.73746
3.125	0.15625	0.84571	0.20532	0.68732	0.51277	0.58526	0.80749
6.25	0.3125	0.93675	0.14231	0.84872	0.43752	0.78971	0.79078
12.5	0.625	0.95175	0.23492	0.87114	0.66727	0.85085	0.78898
25	1.25	0.95805	0.40789	0.94091	0.57803	0.93436	0.64443
50	2.5	0.96962	0.59104	0.96616	0.65782	0.94444	1.08499

Synergy is defined as CI values < 1.0, antagonism as CI values > 1.0, and additivity as CI values = 1.0.

### Effects of Pyrotinib and Adriamycin on the Migration and Invasion of Breast Cancer Cells

**Figure 2:**
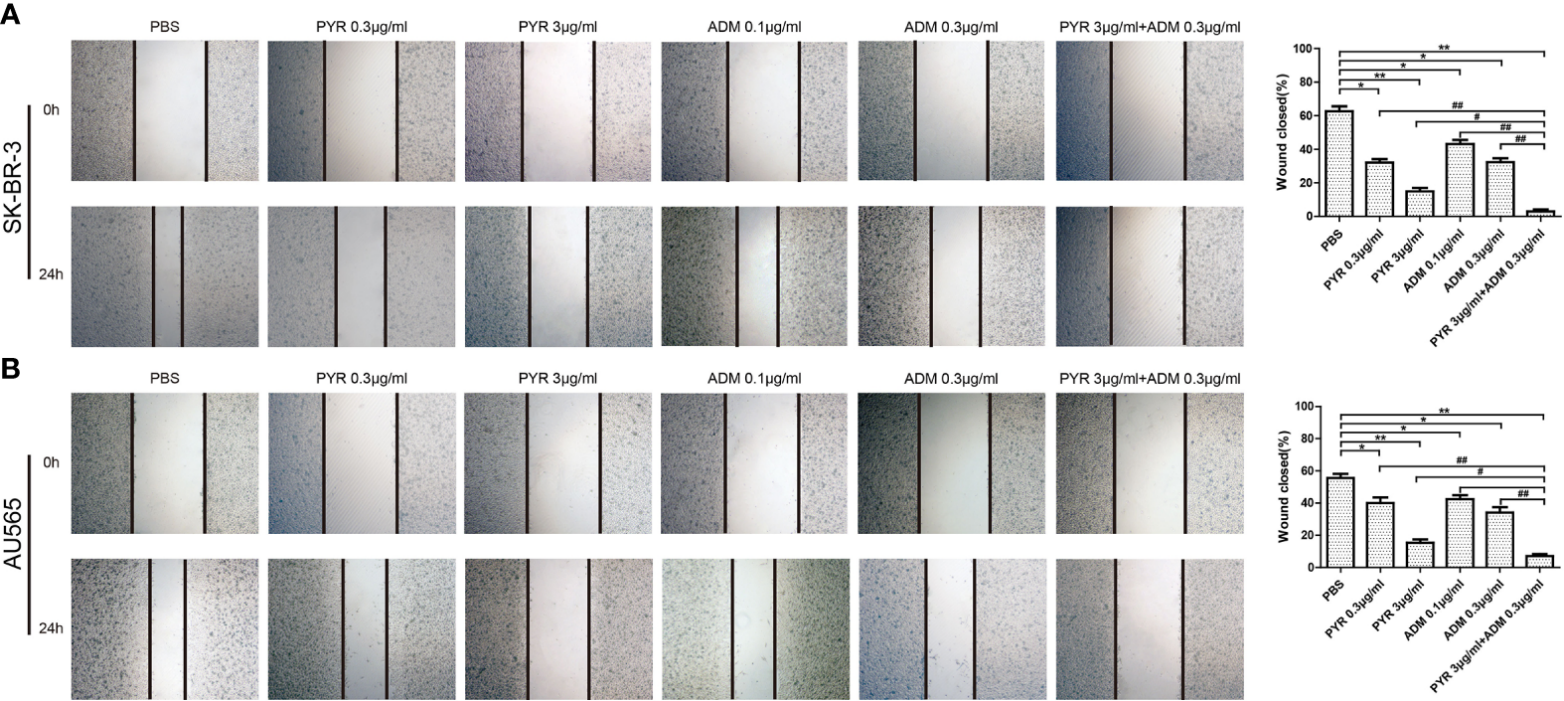
Effects of PYR and ADM on cell migration. The monolayers of SK-BR-3 and AU565 cells were scratched with a pipette tip, and incubated with PBS, different concentrations of PYR (0.3, 3μg/ml), ADM (0.1, 0.3μg/ml) or a combination treatment (PYR+ADM) for 24 h. **(A)** Wound healing assay assessed the effect of PYR and ADM on SK-BR-3 cell migration ability and histogram represents the statistical analysis. **(B)** Wound healing assay assessed the effect of PYR and ADM on AU565 cell migration ability and histogram represents the statistical analysis. Original magnification was ×100. Data represent the mean ± S.D. of three independent experiments. *p<0.05 and **p<0.01 compared with the PBS group, ^#^p<0.05 and ^##^p<0.01 compared with the combination group.

**Figure 3:**
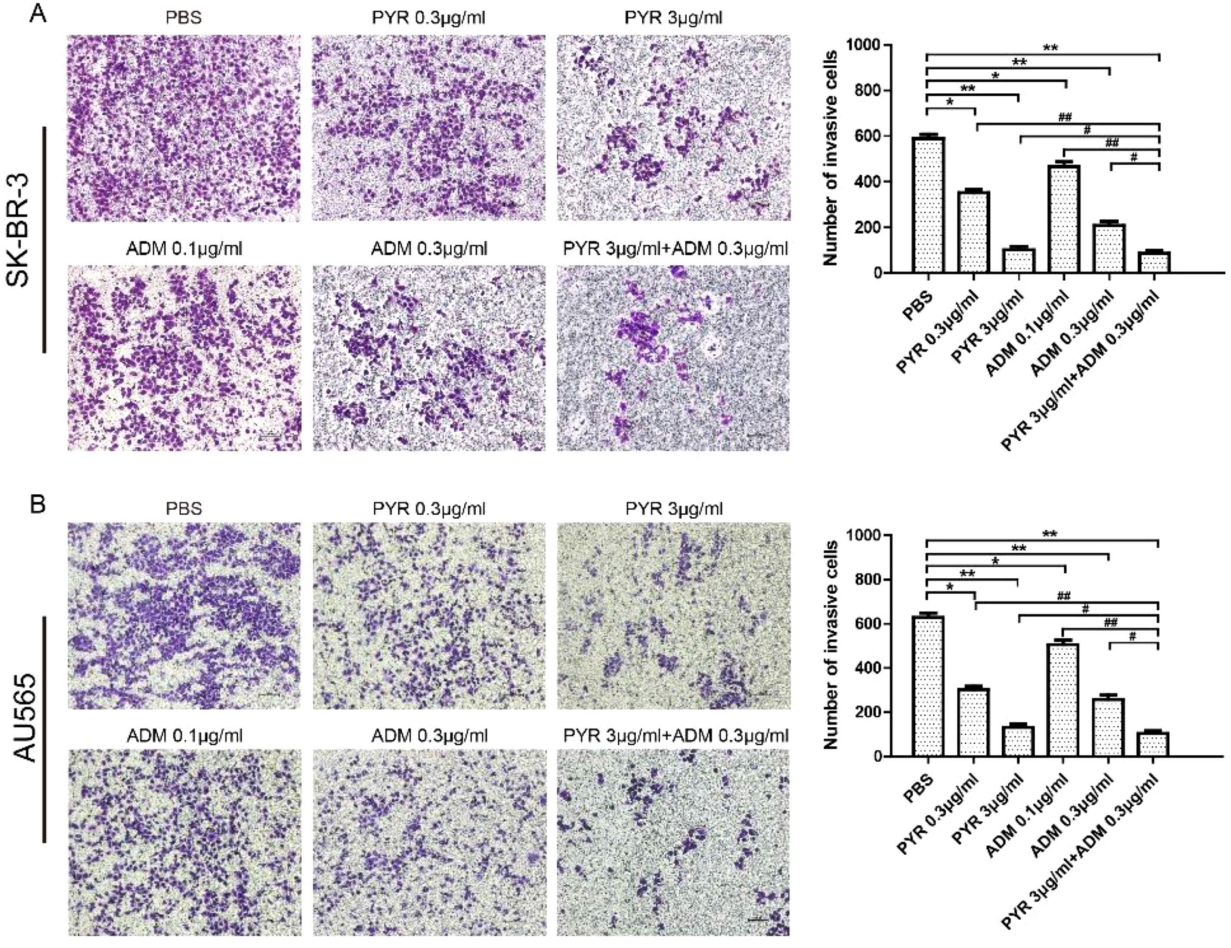
Effects of PYR and ADM on cell invasion. Cell invasion was analyzed with a Matrigel-coated Boyden chamber. SK-BR-3 and AU565 cells were treated with PBS, different concentrations of PYR (0.3, 3μg/ml), ADM (0.1, 0.3μg/ml) or a combination treatment(PYR+ADM) for 24 h. **(A)** Transwell invasion assays assessed the effect of PYR and ADM on SK-BR-3 cell invasion ability and histogram represents the statistical analysis. **(B)** Transwell invasion assays assessed the effect of PYR and ADM on AU565 cell invasion ability and histogram represents the statistical analysis. Original magnification was ×100. Data represent the mean ± S.D. of three independent experiments. *p<0.05 and **p<0.01 compared with the PBS group, ^#^p<0.05 and ^##^p<0.01 compared with the combination group.

The effects of pyrotinib and adriamycin on the migration of SK-BR-3 and AU565 cells were analyzed by wound-healing assays. As shown in **Figure 2**, after treatment with pyrotinib (0.3, 3μg/ml) and adriamycin (0.1, 0.3μg/ml) for 24 h, the migration rate of SK-BR-3 and AU565 cells decreased with the increase of the concentration of pyrotinib and adriamycin (p<0.05), and SK-BR-3 and AU565 cells were treated with both drugs (pyrotinib in combination with adriamycin), the migration rates significantly decreased compared with those cells treated with single drugs (p<0.01). Similar conclusions were found in the transwell invasion assay. The invasion of SK-BR-3 and AU565 cells were dose-dependently inhibited by pyrotinib and adriamycin. The statistical results showed that the number of invasive cells in the pyrotinib and adriamycin group, and decreased remarkably compared with the control group. The inhibitory effect of pyrotinib in combination with adriamycin was more significant than single drugs (p<0.01, **Figure 3**).

### Effects of Pyrotinib and Adriamycin on the Cell Cycle and Apoptosis of Breast Cancer Cells

**Figure 4:**
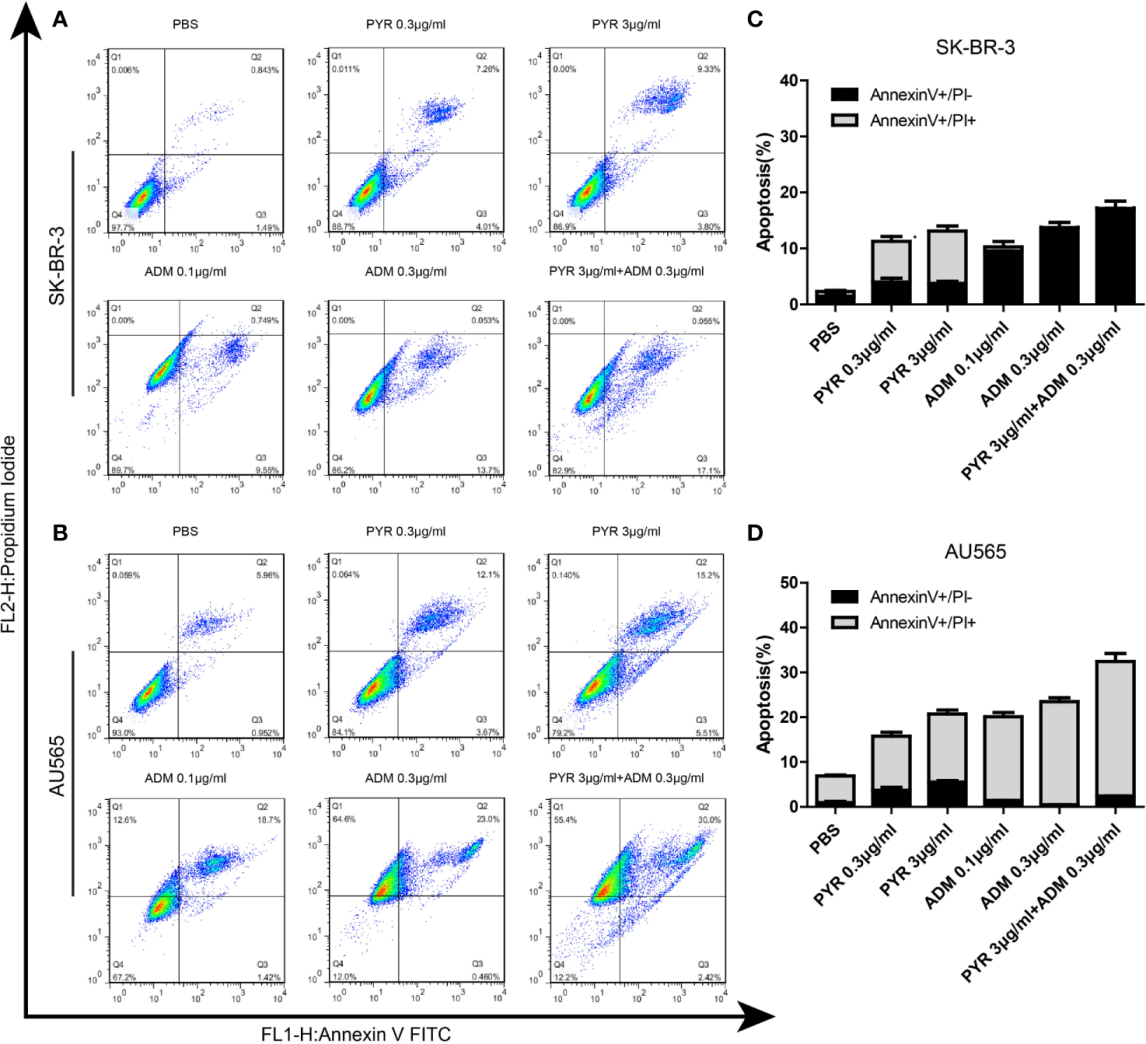
Effects of PYR and ADM on cell apoptosis. **(A, B)** Cell apoptosis was detected through Annexin V-FITC/PI double staining and following flow cytometry for SK-BR-3 cells after incubated with PBS, different concentrations of PYR(0.3, 3μg/ml), ADM(0.1, 0.3μg/ml) or a combination treatment(PYR+ADM) for 24 h. **(C, D)** The histograms were the representative results, while the figure in the right. Compared to the control, PYR combined with ADM induces apoptosis in SK-BR-3 and AU565 cells. Data represent the mean ± S.D. of three independent experiments.

To further investigate the mechanism of the proliferation inhibition on SK-BR-3 and AU565 cells, the effect of pyrotinib and adriamycin on apoptosis was conducted by Annexin V-FITC/PI double staining followed by flow cytometer analysis (**Figure 4**). The increase in the percentage of apoptotic cells was dependent on the increase in concentration. At 24 hours, in SK-BR-3 cells, the early apoptotic cells increased from 7.26% at 0.3 μg/ml to 9.33% at 3 μg/ml of pyrotinib compared to 0.843% of the control group. The number of apoptotic cells increased from 6.12% at 0.1 μg/ml to 8.97% at 0.3 μg/ml of adriamycin compared to 0. 843% of the control group. The percentage of apoptotic cells was 10.2% in the combined group (**Figures 4A, C**). Similarly, in AU565 cells, the early apoptotic cells increased from 5.96% (PBS treated) to 12.1%, 15.2% when cells were treated with 0.3 and 3 μg/ml PYR, respectively. The early apoptotic cells increased from 5.96% (PBS treated) to 18.7%, 23.0% when cells were treated with 0.1 and 0.3 μg/ml ADM, respectively. The percentage of apoptotic cells was 30.00% in the combined group (**Figures 4B, D**).

**Figure 5:**
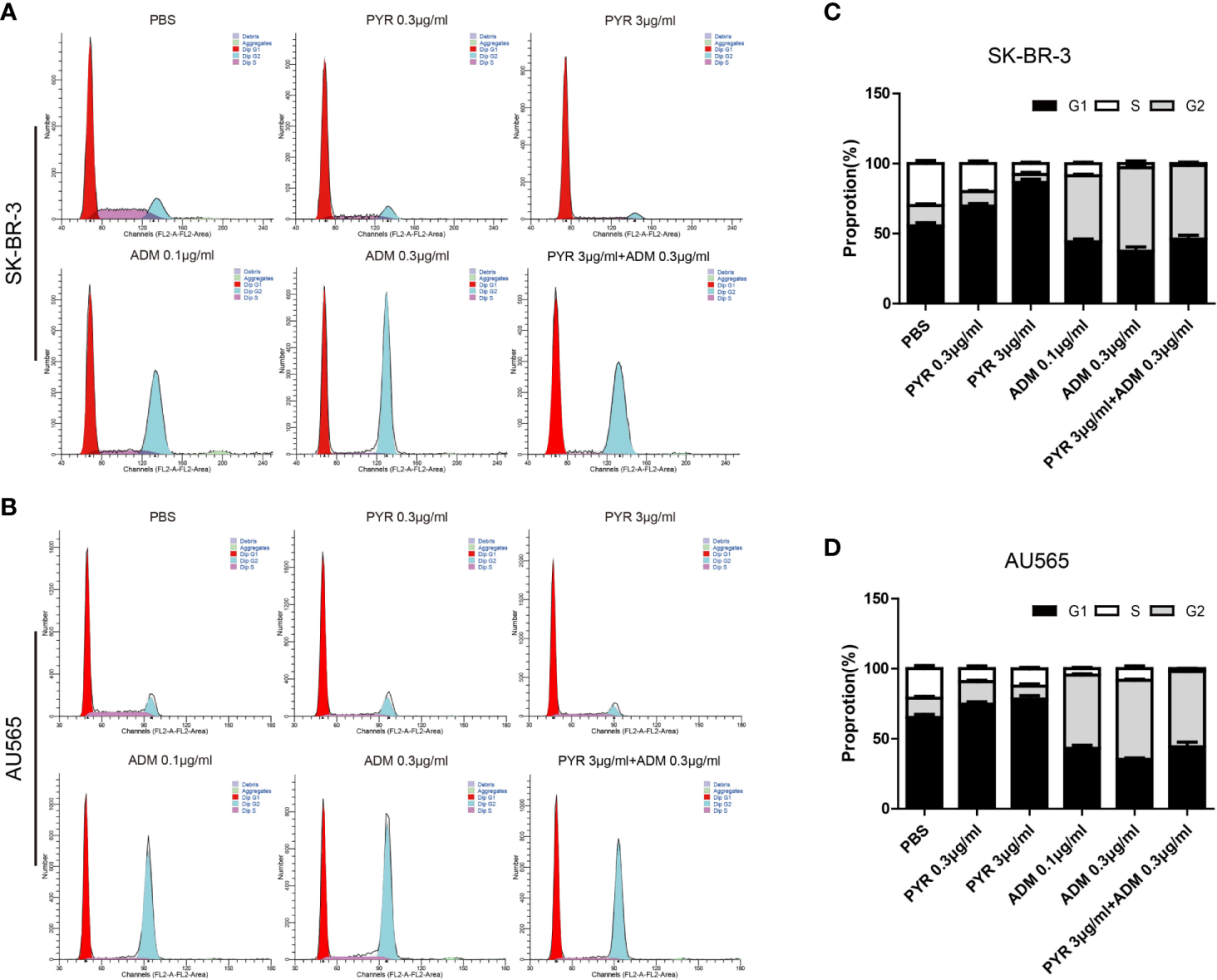
Effects of PYR and ADM on the cell cycle. **(A, B)** Cell cycle analysis through PI staining and following flow cytometry for SK-BR-3 cells after incubated with PBS, different concentrations of PYR(0.3, 3μg/ml), ADM(0.1, 0.3μg/ml) or a combination treatment (PYR+ADM) for 24 h. ModFit was used to perform cell cycle analysis. **(C, D)** The histograms were the representative results, while the figure in the right. Compared with the PBS group, PYR caused significant G1 phase arrest, while ADM caused significant G2 phase arrest, PYR combined with ADM induces G1/S arrest in SK-BR-3 and AU565 cells. Data represent the mean ± S.D. of three independent experiments.

The distribution of the cell cycle phase in SK-BR-3 and AU565 cells treated with pyrotinib and adriamycin at 24h was depicted in **Figure 5**. The cell cycle arrest by pyrotinib and adriamycin was concentration-dependent. Compared with the untreated control group, treatment with pyrotinib resulted in the significant cell stagnation of SK-BR3 and AU565 cells in the G1 phase of the cell cycle. On the other hand, adriamycin treatment makes SK-BR3 and AU565 cell cycles arrest in the G2 phase. As shown in **Figure 5**, compared to the PBS group, the percentage of SK-BR-3 and AU565 cells arrested in the G2 phase was increased in the combination group.

### *In Vivo* Anticancer Effect of Pyrotinib and Adriamycin in SK-BR-3 BC Xenograft Models

**Figure 6:**
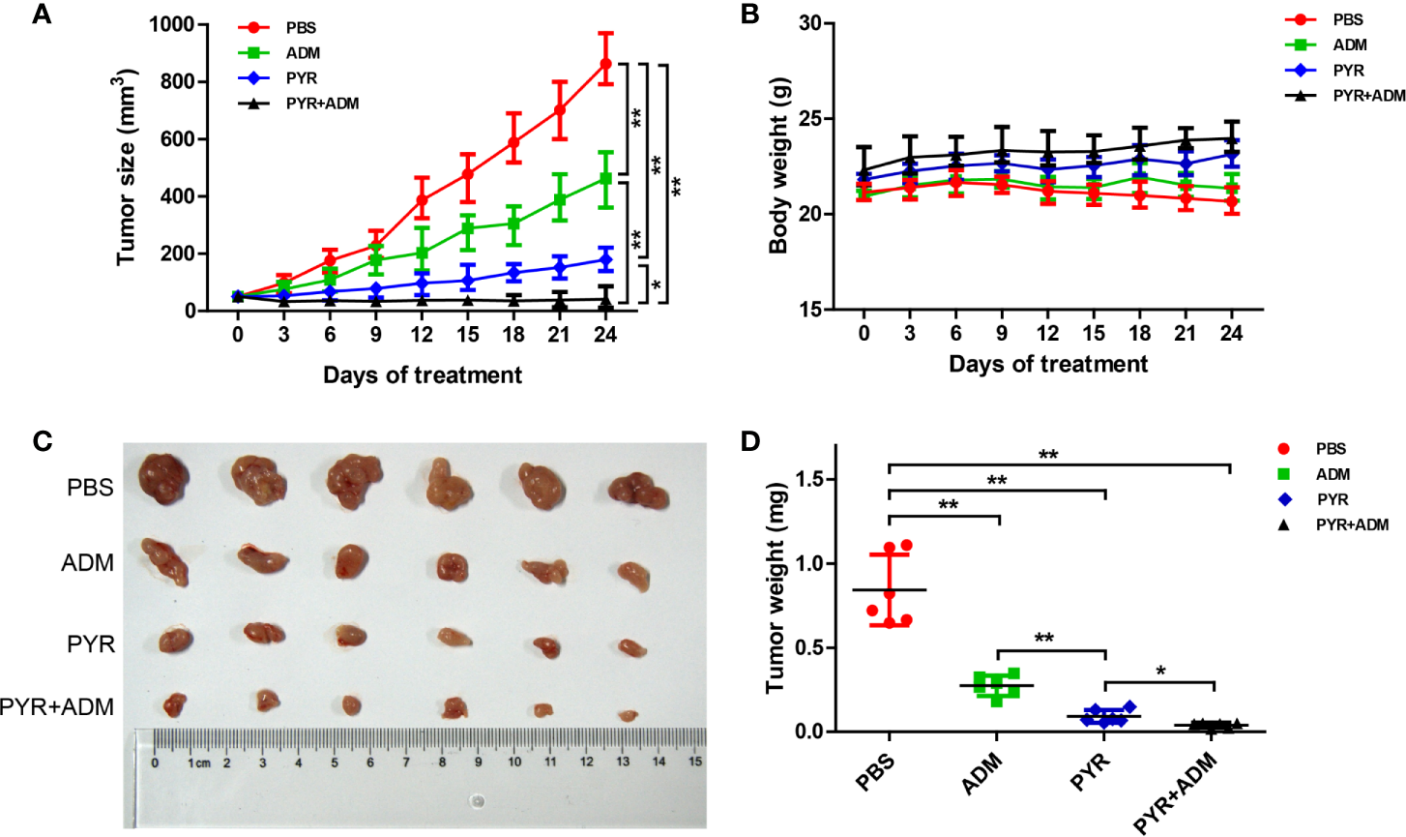
In vivo anticancer effect of PYR and ADM in breast cancer xenograft models. Randomly grouped nude mice were treated with PBS, PYR(30 mg/kg), ADM(5 mg/kg), or a combination treatment (PYR+ADM) for 27 days. **(A, B)** Tumor growth ratio curve and body weight changes every three days after the onset of treatment. **(C, D)** Photos of the excised tumors and weight obtained on day 27 after treatment. *p<0.05 and **p<0.01.

After confirming the inhibitory effect of pyrotinib combined with adriamycin on cell proliferation and cell growth *in vitro*, The tumor model was established to further evaluate the inhibitory effect of the two drugs on tumor growth either individually or synergistically. Mice with SK-BR-3 xenografts were randomly divided into 4 treatment groups. These groups included the PBS control group, pyrotinib (30 mg/kg every day), adriamycin (5 mg/kg weekly), or the combination of pyrotinib (30 mg/kg every day) and adriamycin (5 mg/kg weekly). The relative tumor volume and body weight were measured twice per week. The results showed that pyrotinib was more effective than adriamycin in inhibiting tumor growth (p<0.05, **Figures 6A, C, D**). Nevertheless, the combined drug group (pyrotinib and adriamycin) had a stronger inhibitory effect on SK-BR-3 xenograft growth than any drug alone (**Figures 6A, C, D**). All the treatments caused a significant inhibition in tumor growth as compared with the PBS control group (p<0.05).

No general toxicities were noted in all groups, as all groups showed a slightly steady increase in body weight without significant difference (**Figure 6B**). These data indicate that the combination of pyrotinib and adriamycin can augment anticancer activity without increased toxicity.

### Effects of Pyrotinib and Adriamycin on the Expression of Akt, p-Akt, p65, p-p65 and FOXC1

**Figure 7:**
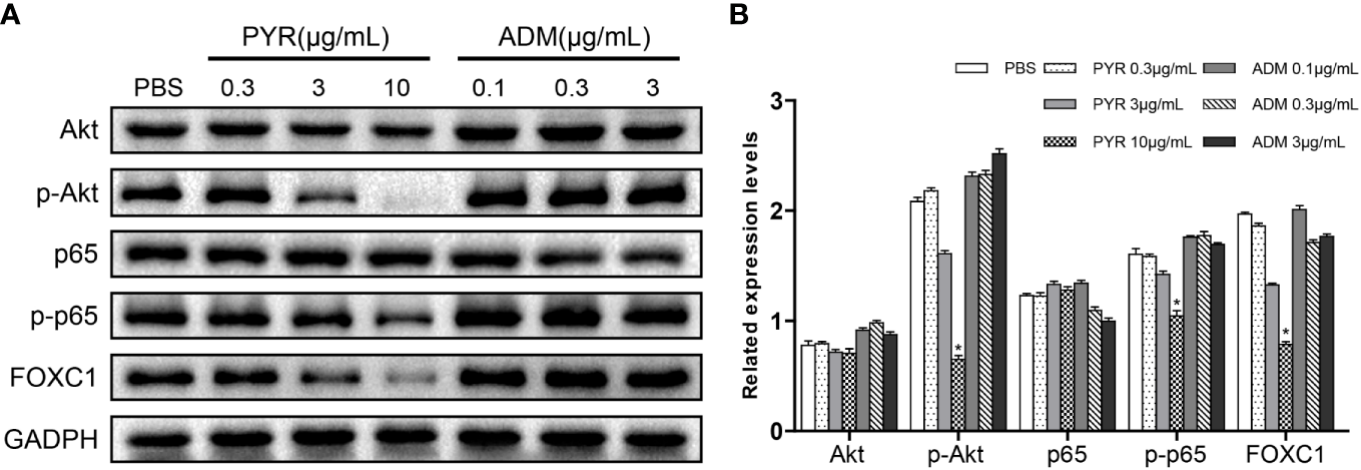
Molecular mechanism studies in breast cancer cells after treatment of PBS, PYR, ADM, or a combined treatment. SK-BR-3 cells were treated with PBS, different concentrations of PYR (0.3, 3, 10μg/ml), ADM (0.1, 0.3, 3μg/ml) for 24 h, respectively. Nuclear and cytosolic protein extracts were subjected to Western blot analysis. **(A)** The results of Western blot for Akt, p-Akt, p65, p-p65, and FOXC1 in the nuclear fractions and cytosolic extracts, respectively. GADPH served as the loading control. **(B)** Quantitative analysis of the Western blotting results. Data represent the mean ± S.D. of three independent experiments. *p<0.05 with the PBS group.

To investigate the mechanism of pyrotinib and adriamycin on BC cells, Western blots were used to assess the protein content of SK-BR-3 cells after treatment with PBS, pyrotinib (0.3, 3, 10μg/ml) or adriamycin (0.1, 0.3, 3μg/ml). We evaluated several key molecules in cell signaling pathways. The results showed that p-Akt, p-p65, FOXC1 activity were tremendously decreased by pyrotinib treatment at 3, 10μg/ml after 48 h incubation, whereas no significant effect on p-Akt, p-p65, FOXC1 activity was observed in the adriamycin group (**Figure 7**).

### Antitumor Activity of Pyrotinib in BC Patients

**Figure 8:**
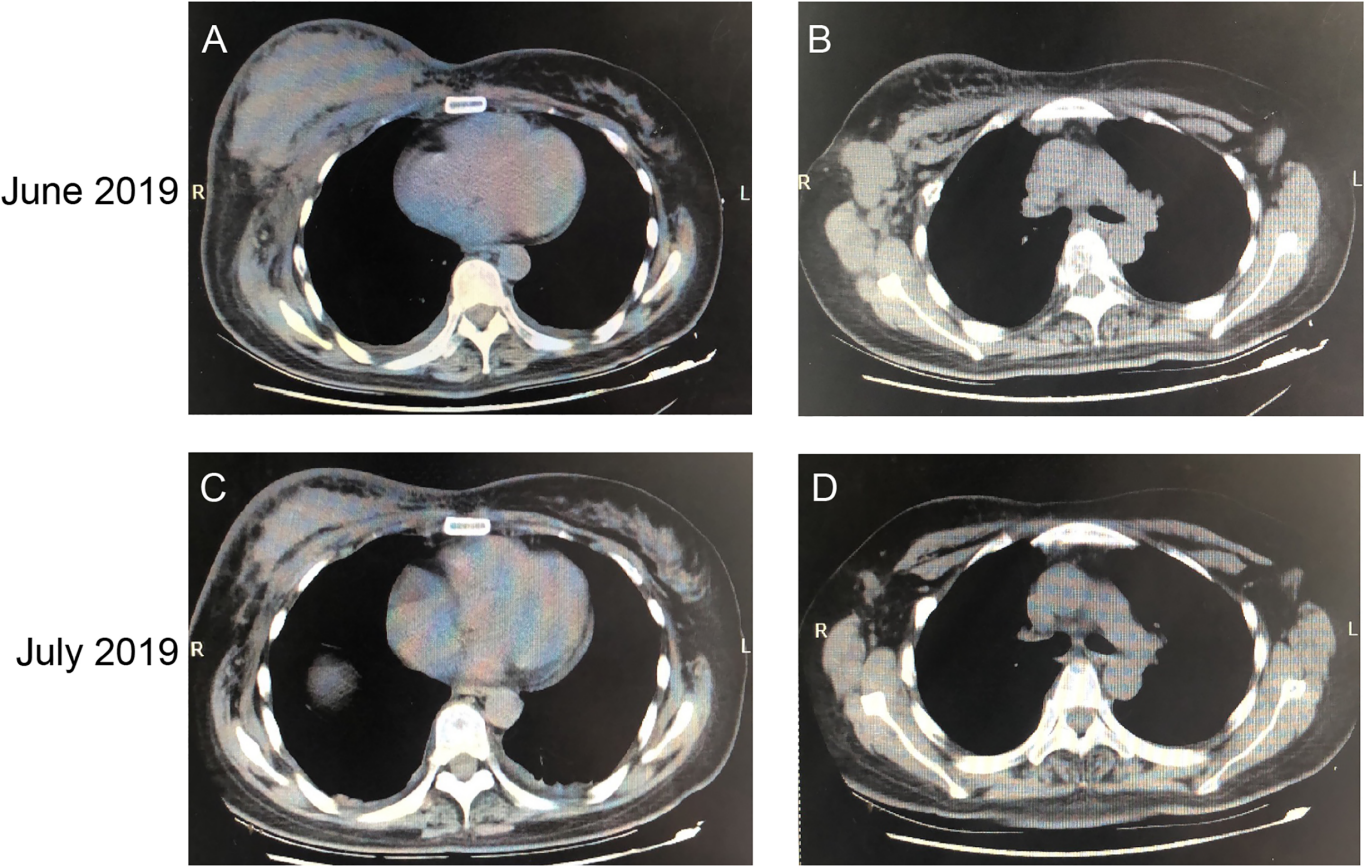
CT Imaging of the breast and lymph node before and after pyrotinib treatment. CT scans before the administration of pyrotinib (images **A**, **B**) and after one month of treatment (images **C**, **D**), showing the tumor mass and lymph node.

A 52-year-old female patient was clinically diagnosed with right breast cancer and lymph node metastasis in 2017. The histopathological diagnosis was HER2 amplification, ER negative, PR negative, Ki-67 20%. Subsequently, the patient received chemotherapy and trastuzumab treatment. However, the patient still suffered from disease progression after multiline treatment. As shown in **Figures 8A, B**, breast and lymph node had progressed significantly. Since June 16, 2019, the patient has been taking 400 mg of pyrotinib orally on a daily basis. In July 2019, the chest CT showed that the right breast lump and lymph node were all smaller than before (**Figures 8C, D**). The efficacy evaluation confirmed partial response (PR) compared with the baseline computed CT scan.

## Discussion

Breast cancer is the most frequently diagnosed cancer and the leading cause of cancer-related death among women. HER2 is overexpressed in about 15-20% of breast cancer patients. This transmembrane receptor tyrosine kinase promotes abnormal cell growth and proliferation in human breast cancer, resulting in aggressive tumor cells and poor prognosis. With the further understanding of the molecular mechanism of HER2-positive breast cancer, a series of HER2-targeted drugs have been developed, including trastuzumab, pertuzumab, lapatinib, neratinib, T-DM1, and pyrotinib have been approved for the treatment of HER2-positive breast cancer.

Pyrotinib is an irreversible dual pan-ErbB receptor tyrosine kinase inhibitor developed for the treatment of HER2-positive advanced malignant solid tumors. In August 2018, the Chinese State Drug Administration first conditionally approved pyrotinib for use in combination with capecitabine for the treatment of HER2-positive, advanced or metastatic breast cancer in patients previously treated with anthracycline or taxane chemotherapy (11). In a randomized, open, controlled I/II clinical study, the efficacy and safety of pyrotinib plus capecitabine in contrast with lapatinib plus capecitabine were evaluated in the treatment of HER2-positive recurrent or metastatic BC. Compared with lapatinib combined with capecitabine, pyrotinib combined with capecitabine has a higher objective response rate (79 vs. 57%; p = 0.01). Pyrotinib combined with capecitabine significantly prolonged median progression-free survival versus lapatinib combined with capecitabine (18.1 vs. 7.0 months p<0.0001) (11, 36). The result of the phase III trial assessing pyrotinib versus placebo both in combination with capecitabine in women with HER2-positive metastatic BC who received prior taxanes and trastuzumab therapy was reported at ASCO in June 2019. Patients were randomly assigned to be administrated with pyrotinib plus capecitabine (*n*=185) or placebo plus capecitabine (n=94). The median PFS for the combination group was 11.1 months, and that for the placebo group was 4.1 months. Furthermore, 71 patients in the placebo group whose disease progressed received pyrotinib monotherapy afterward, revealed a single drug response rate of 38.0% and the median PFS of 5.5 months (41). It is anticipated that combining pyrotinib with chemotherapy will be a trend for anti-HER2 therapy in the future. 

In this study, we examined the effect of pyrotinib and adriamycin on cell lines and xenograft models. MTT assay and flow cytometry showed that pyrotinib and adriamycin significantly inhibited the growth of the breast cancer cell line and induced cell apoptosis in a concentration-dependent manner. We also observed that co-treatment with pyrotinib and adriamycin led to growth inhibition in breast cancer cells. Although previous studies revealed that pyrotinib displayed cytotoxic effects and induced apoptosis in breast cancer cell lines *via* different molecular mechanisms, there is no research on the effect of pyrotinib on HER2-positive breast cancer cell migration and invasion. The present study found that pyrotinib and adriamycin significantly inhibited cell migration and invasion in the breast cancer cells, which has a strong ability in cell migration and invasion. Furthermore, the inhibitory effect of pyrotinib was more significant than adriamycin.

The combined effect of pyrotinib and adriamycin was demonstrated *in vivo*, the results indicated that the anticancer effect of the combinatorial treatment was higher than any other single drug, which was consistent with *in vitro*. In addition, nude mice were treated with combinatorial treatment but did not show worse body weight than the patients in the groups treated with pyrotinib or adriamycin alone (**Figure 6B**). Hence, our data show that a combination of pyrotinib and adriamycin led to enhanced antitumor activity without extended toxicity. To evaluate the efficacy of pyrotinib treatment in a clinical setting, we observed the effect of HER2-positive breast cancer patients before and after treatment with pyrotinib, the results show that pyrotinib also has good antitumor activity (**Figure 8**). Nevertheless, more clinical trials are needed to confirm the efficacy of pyrotinib on HER2-positive breast cancer.

**Figure 9:**
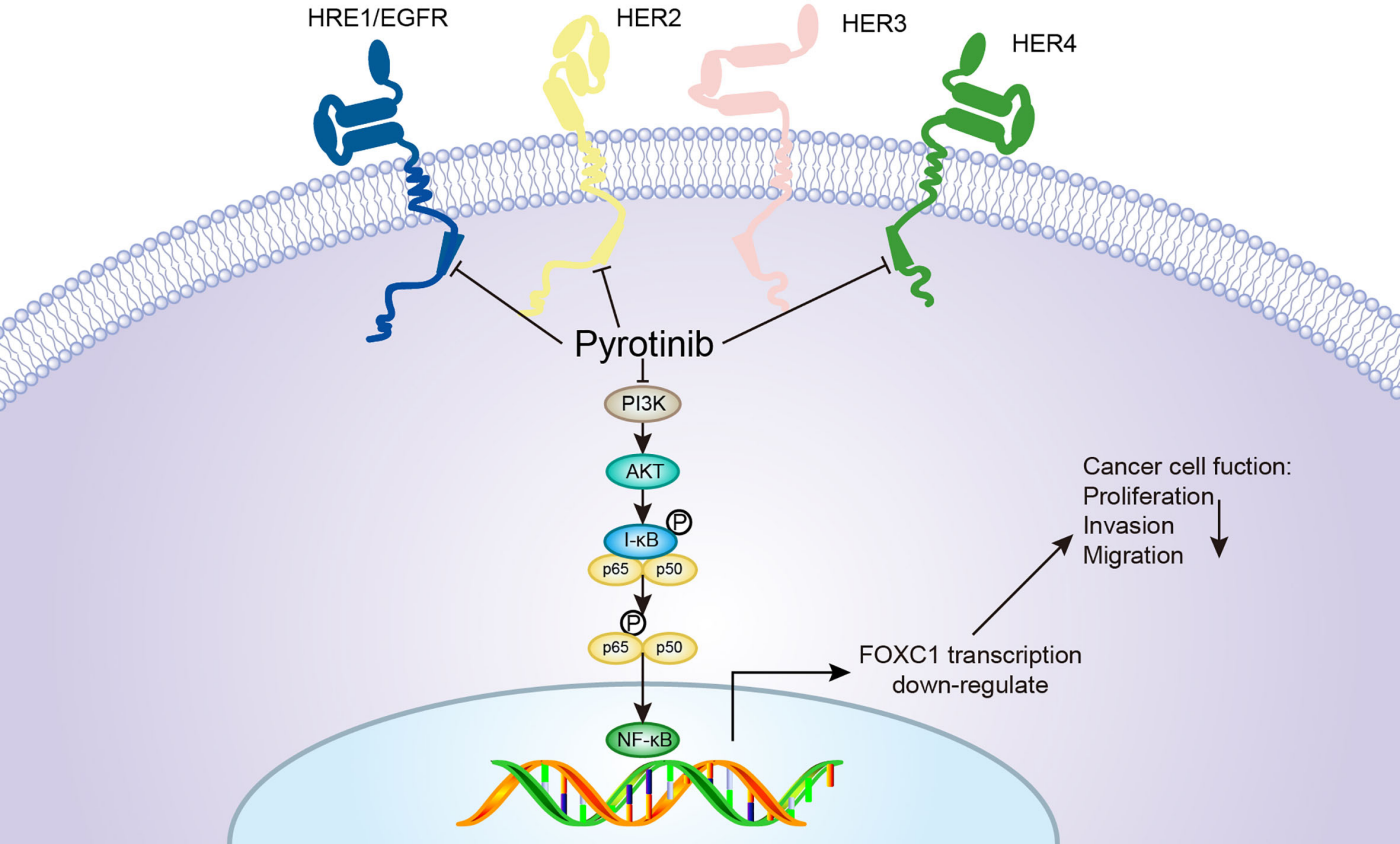
A proposed model for the pyrotinib-mediated inhibitory effects on the apoptosis, migration, and invasion of BCs. Pyrotinib is an irreversible HER 1, 2, and 4 inhibitor that inhibits the downstream signals of the Akt/p-65/FOXC1 pathway, resulting in a significant efficacy in cell proliferation, migration, and invasion of HER2-overexpressing cancer cells.

To further explore the potential molecular mechanisms and signaling pathways involved in the anticancer effects of pyrotinib and adriamycin, western blotting was used to evaluate molecular changes upon pyrotinib or adriamycin therapy. We found that pyrotinib treatment significantly down-regulated Akt, p-65 phosphorylation and reduced the protein level of FOXC1 in the breast cancer cells, but adriamycin had no significant effect on the expression and phosphorylation of Akt, p65, and FOXC1 in breast cancer cells. These findings indicate that pyrotinib inhibited cell proliferation, migration, and invasion possibly through inactivation of Akt/p-65/FOXC1 signaling in HER2-positive breast cancer cells. Consistent with our findings, Zhang et al. also reported that pyrotinib treatment down-regulated Akt phosphorylation in breast cancer cells (31). Previous studies also showed that NF-κB-p65 enhances FOXC1 promoter activity in basal-like breast cancer cells (MDA-MB-468) (42). According to our findings, we proposed a schematic presentation of possible mechanisms for the suppressive effects of pyrotinib on proliferation, migration, and invasion in HER2-positive breast cancer cells (**Figure 9**).

In summary, this is the first work to reveal the antitumor activity of pyrotinib combined with adriamycin on HER2-positive breast cancer *in vitro* and *in vivo*, which may be more effective than pyrotinib or adriamycin alone. This study reveals the molecular mechanisms of pyrotinib in the treatment of breast cancer, which provides a theoretical basis for the comprehensive treatment of breast cancer in clinics.

## Data Availability Statement

The raw data supporting the conclusions of this article will be made available by the authors, without undue reservation.

## Ethics Statement

The studies involving human participants were reviewed and approved by Ethics Committee of the First Affiliated Hospital of Henan University of Science and Technology. The patients/participants provided their written informed consent to participate in this study. The animal study was reviewed and approved by Ethics Committee of the First Affiliated Hospital of Henan University of Science and Technology.

## Author Contributions

CW and XW designed and carried out the experiments. SD and XY analyzed the data. CW and YL wrote the manuscript. JC, XX, and XH performed MTT assay, wound-healing, and transwell invasion assays, flow cytometry, and Western blotting assays. CY and FS collected the clinical samples. DK and GL performed animal models. XW provided supervision and guidance. All authors contributed to the article and approved the submitted version.

## Funding

This work was supported in part by grants from the project of the Science and Technology Department of Henan Province (2018010019).

## Conflict of Interest

The authors declare that the research was conducted in the absence of any commercial or financial relationships that could be construed as a potential conflict of interest.
