# Corrigendum: The synergistic effects of pyrotinib combined with adriamycin on HER2-positive breast cancer

**DOI:** 10.3389/fonc.2025.1536374

**Published:** 2025-03-18

**Authors:** Chaokun Wang, Shuzhen Deng, Jing Chen, Xiangyun Xu, Xiaochen Hu, Dejiu Kong, Gaofeng Liang, Xiang Yuan, Yuanpei Li, Xinshuai Wang

**Affiliations:** ^1^ Henan Key Laboratory of Cancer Epigenetics, Cancer Hospital, The First Affiliated Hospital, College of Clinical Medicine, Medical College of Henan University of Science and Technology, Luoyang, China; ^2^ Medical College, Henan University of Science and Technology, Luoyang, China; ^3^ Department of Internal Medicine, UC Davis Comprehensive Cancer Center, University of California Davis, Sacramento, CA, United States

**Keywords:** HER2 positive breast neoplasm, pyrotinib, adriamycin, synergistic, Akt

In the published article, there was an error in **Figure 3** as published. Due to the large number of photos taken, careless naming errors led to the accidental reuse of some images. The corrected **Figure 3** and its caption **Figure 3**. Effects of PYR and ADM on cell invasion appear below.

**Figure 3:**
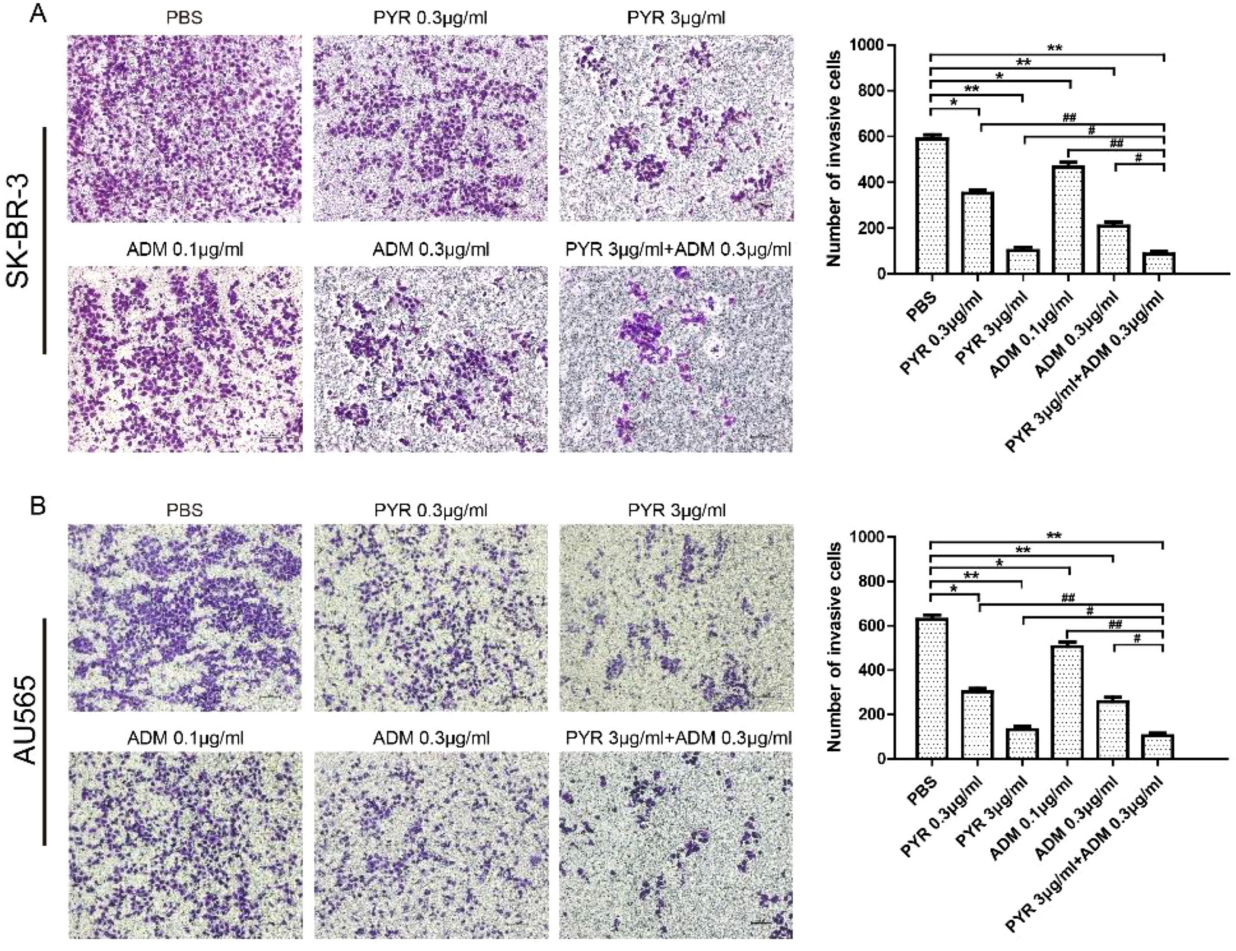
Effects of PYR and ADM on cell invasion. Cell invasion was analyzed with a Matrigel-coated Boyden chamber. SK-BR-3 and AU565 cells were treated with PBS, different concentrations of PYR (0.3, 3μg/ml), ADM (0.1, 0.3μg/ml) or a combination treatment(PYR+ADM) for 24 h. **(A)** Transwell invasion assays assessed the effect of PYR and ADM on SK-BR-3 cell invasion ability and histogram represents the statistical analysis. **(B)** Transwell invasion assays assessed the effect of PYR and ADM on AU565 cell invasion ability and histogram represents the statistical analysis. Original magnification was ×100. Data represent the mean ± S.D. of three independent experiments. *p<0.05 and **p<0.01 compared with the PBS group, ^#^p<0.05 and ^##^p<0.01 compared with the combination group.

The authors apologize for this error and state that this does not change the scientific conclusions of the article in any way. The original article has been updated.

